# Crystal structure of betulinic acid methanol monosolvate

**DOI:** 10.1107/S1600536814023848

**Published:** 2014-11-08

**Authors:** Wei Tang, Neng-Hua Chen, Guo-Qiang Li, Guo-Cai Wang, Yao-Lan Li

**Affiliations:** a Guangdong Province Key Laboratory of Pharmacodynamic Constituents of Traditional Chinese Medicine and New Drugs Research, Institute of Traditional Chinese Medicine and Natural Products, College of Pharmacy, Jinan University, Guangzhou 510632, People’s Republic of China

**Keywords:** crystal structure, betulinic acid, lup-20(29)-en-28-oic acid, *Syzygium jambos* (L.) Alston, hydrogen bonding, natural product

## Abstract

The title compound [systematic name: 3β-hy­droxy­lup-20(29)-en-28-oic acid methanol monosolvate], C_30_H_48_O_3_·CH_3_OH, is a solvent pseudopolymorph of a naturally occurring plant-derived lupane-type penta­cyclic triterpenoid, which was isolated from the traditional Chinese medicinal plant *Syzygium jambos* (L.) Alston. The dihedral angle between the planes of the carb­oxy­lic acid group and the olefinic group is 12.17 (18)°. The *A/B*, *B*/*C*, *C*/*D* and *D*/*E* ring junctions are all *trans*-fused. In the crystal, O—H⋯O hydrogen bonds involving the hy­droxy and carb­oxy­lic acid groups and the methanol solvent mol­ecule give rise to a two-dimensional network structure lying parallel to (001).

## Related literature   

For general background to the synthesis, extraction and pharmceutical activities of the title compound, see: Kashiwada *et al.* (1996 ▶); Fulda *et al.* (1999 ▶); Liu *et al.* (2009 ▶); Safe *et al.* (2012 ▶); Babalola *et al.* (2013 ▶); Heidary Navid *et al.* (2014 ▶); Yadav & Gupta (2014 ▶). For the structure of another methanol solvate of betulinic acid, see: Wang *et al.* (2014 ▶).
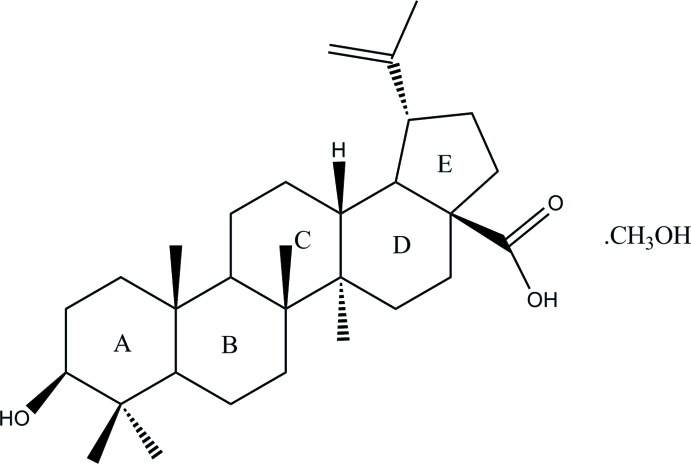


## Experimental   

### Crystal data  

C_30_H_48_O_3_·CH_4_O
*M* *_r_* = 488.73
Orthorhombic, 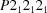

*a* = 7.0988 (2) Å
*b* = 12.3864 (3) Å
*c* = 33.2745 (9) Å
*V* = 2925.78 (13) Å^3^
*Z* = 4
Cu *K*α radiation
μ = 0.55 mm^−1^
*T* = 293 K
0.28 × 0.25 × 0.20 mm

### Data collection  

Oxford Diffraction Gemini S Ultra CCD-detector diffractometer
Absorption correction: multi-scan (*CrysAlis PRO*; Agilent, 2011 ▶) *T*_min_ = 0.748, *T*_max_ = 1.000
8319 measured reflections
4343 independent reflections
3796 reflections with *I* > 2σ(*I*)
*R*_int_ = 0.030

### Refinement  

*R*[*F* ^2^ > 2σ(*F* ^2^)] = 0.044
*wR*(*F* ^2^) = 0.112
*S* = 1.05
4343 reflections
326 parameters
H-atom parameters constrained
Δρ_max_ = 0.15 e Å^−3^
Δρ_min_ = −0.19 e Å^−3^

### 

Data collection: *CrysAlis PRO* (Agilent, 2011 ▶); cell refinement: *CrysAlis PRO*; data reduction: *CrysAlis PRO*; program(s) used to solve structure: *SHELXS97* (Sheldrick, 2008 ▶); program(s) used to refine structure: *SHELXL97* (Sheldrick, 2008 ▶); molecular graphics: *OLEX2* (Dolomanov *et al.*, 2009 ▶); software used to prepare material for publication: *OLEX2*.

## Supplementary Material

Crystal structure: contains datablock(s) I, global. DOI: 10.1107/S1600536814023848/zs2317sup1.cif

Structure factors: contains datablock(s) I. DOI: 10.1107/S1600536814023848/zs2317Isup2.hkl

**Fig. 1.**
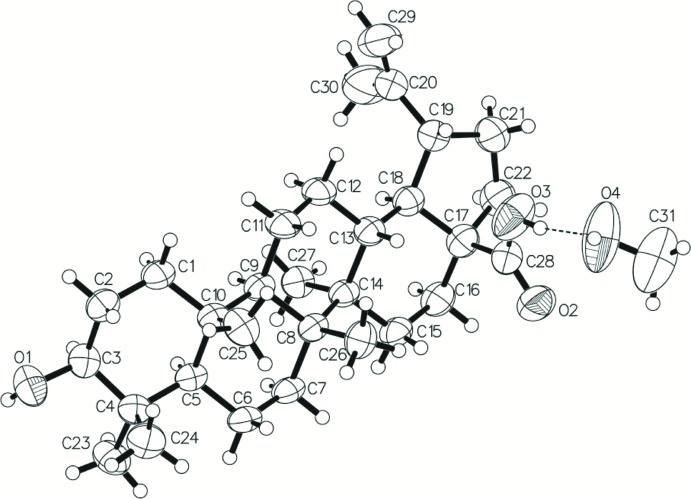
The molecular structure of the title compound showing 50% probability displacement ellipsoids and the atom-numbering scheme.

. DOI: 10.1107/S1600536814023848/zs2317fig1.tif
The mol­ecular structure of the title compound showing 50% probability displacement ellipsoids and the atom-numbering scheme.

CCDC reference: 1031558

Additional supporting information:  crystallographic information; 3D view; checkCIF report

## Figures and Tables

**Table 1:** Hydrogen-bond geometry (, )

*D*H*A*	*D*H	H*A*	*D* *A*	*D*H*A*
O3H3*A*O4	0.82	1.76	2.571(3)	170
O1H1O2^i^	0.82	1.95	2.753(3)	165
O4H4O1^ii^	0.82	1.83	2.640(3)	168

Symmetry codes: (i) 
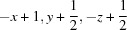
; (ii) 
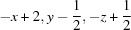
.

## supplementary crystallographic information

### S1. Comment

The title compound C_30_H_48_O_3_.CH_3_OH (Fig. 1) is a lupane-type pentacyclic triterpenoid [systematic name: 3β-hydroxy-lup-20 (29)-en-28-oic acid], which is a natural product isolated from plants of, e.g. *Betula spp*., *Diospyros spp*., *Paeonia spp*., *Sambucus spp*., *Syzygium spp*. and *Ziziphus spp*. (Wang *et al.*, 2014) but mainly from the bark of *Betula pubescens*. It also may be obtained from the partial synthesis of betulin or preparation by biological fermentation with betulin. Betulinic acid has been demonstrated to have very high pharmacological values (Liu *et al.*, 2009), such as anti-HIV (Kashiwada *et al.*, 1996), anti-HSV-1 (Heidary *et al.*, 2014), anti-tumor (Fulda *et al.*, 1999), anti-platelet-aggregation (Babalola *et al.*, 2013) and anti-cancer activities (Safe *et al.*, 2012), together with anti-inflammatory (Yadav & Gupta, 2014), and antimalarial activities (Wang *et al.*, 2014).

Five crystalline pseudopolymorphic forms of betulinic acid have been reported, including a triclinic methanol solvate (space group *P*1 with *Z* = 1), obtained from a saturated methanolic solution (Wang *et al.*, 2014). The title compound, the methanol monosolvate C_30_H_48_O_3_ . CH_3_OH represents another pseudopolymorph which crystallizes in the orthorhombic space group *P*2_1_2_1_2_1_ with *Z* = 4. This compound (Fig. 1) is composed of five rings (*A*–*E*), one five-membered *E* the others six-membered. The five-membered ring adopts a boat conformation, which has puckering parameters, φ = 0.2 (4)°. The six-membered ring *A* adopts a chair conformation with puckering parameters Q = 0.546 (3)Å, θ = 2.7 (3)°, φ = 100 (4)°. Rings *B*, *C* and *D* adopt chair conformations with puckering parameters Q = 0.575 (2)Å, θ = 10.9 (2)°,φ = 3.6 (12)°; Q = 0.607 (2)Å, θ = 8.12 (19)°, φ = 330.0 (16)°; Q = 0.580 (2)Å, θ = 170.7 (2)°, φ = 88.9 (15)Å, respectively. The *A/B*, *B/C*, *C/D* and *D/E* ring junctions are all *trans*-fused. The dihedral angle between the planes of the carboxylic group and the olefinic group is 12.17 (18)°.

In the crystal, an intermolecular hydroxy O1—H···O2^i^_carboxyl_ hydrogen bond (Table 1) links the betulinic acid molecules into a zig-zag chain which extends along *b*. The methanol solvent molecule is linked to the parent molecule by a carboxylic acid O3—H···O4_methanol_ hydrogen bond while the methanol molecule extends the structure through an O4—H···O1^ii^ interaction, giving a two-dimensional network structure lying parallel to (001). The absolute configuration determined for betulinic acid (Wang *et al.*, 2014) was invoked, giving the assignments C3(*S*),C5(*R*),C8(*R*),C9(*R*), C10(*R*), C13(*R*),C14(*R*),C17(*S*),C18(*R*), C19(*R*) for the 10 chiral centres in the molecule (using the arbitrary atom numbering scheme employed in Fig.1.

### S2. Experimental

The title compound was isolated from the herbs of the traditional Chinese medicine *Syzygium jambos* (L.) Alston. The herbs of *Syzygium jambos* (L.) Alston (5 kg) was extracted with 95% ethanol at room temperature and the extracted solution was concentrated by rotary evaporator. The crude extract was suspended in distilled water and partitioned with petroleum ether, ethyl acetate and *n*-butanol. The title compound (50 mg) was isolated from the petroleum ether fraction through silica gel column chromatography and crystals were obtained after slow evaporation of a saturated methanol solution at room temperature.

### S3. Refinement

All H atoms were positioned geometrically and were included in the refinement in the riding-model approximation, with C—H = 0.96 Å (CH_3_) and *U*_iso_(H) = 1.5*U*_eq_(C); 0.97 Å (CH_2_) and *U*_iso_(H) = 1.2*U*_eq_(C); 0.93 Å (aryl H) and *U*_iso_(H)= 1.2*U*_eq_(C); O—H = 0.82 Å and *U*_iso_(H) = 1.5*U*_eq_(O). The configuration of the 10 chiral centres in the betulinic acid [C3(*S*),C5(*R*),C8(*R*),C9(*R*), C10(*R*), C13(*R*),C14(*R*),C17(*S*),C18(*R*), C19(*R*)] was invoked, giving a Flack parameter of 0.3 (3) for 1624 Friedel pairs (Flack, 1983) for the arbitrary atom numbering scheme used in this article.

### Crystal data

**Table:**

C_30_H_48_O_3_·CH_4_O	*D*_x_ = 1.110 Mg m^−^^3^
*M**_r_* = 488.73	Cu *K*α radiation, λ = 1.54184 Å
Orthorhombic, *P*2_1_2_1_2_1_	Cell parameters from 3107 reflections
*a* = 7.0988 (2) Å	θ = 3.8–62.4°
*b* = 12.3864 (3) Å	µ = 0.55 mm^−^^1^
*c* = 33.2745 (9) Å	*T* = 293 K
*V* = 2925.78 (13) Å^3^	Block, colourless
*Z* = 4	0.28 × 0.25 × 0.20 mm
*F*(000) = 1080	

### Data collection

**Table:**

Oxford Diffraction Gemini S Ultra CCD-detector diffractometer	4343 independent reflections
Radiation source: Enhance Ultra (Cu) X-ray source	3796 reflections with *I* > 2σ(*I*)
Mirror monochromator	*R*_int_ = 0.030
Detector resolution: 16.0288 pixels mm^-1^	θ_max_ = 62.8°, θ_min_ = 3.8°
ω scans	*h* = −8→6
Absorption correction: multi-scan (*CrysAlis PRO*; Agilent, 2011)	*k* = −14→14
*T*_min_ = 0.748, *T*_max_ = 1.000	*l* = −38→30
8319 measured reflections	

### Refinement

**Table:**

Refinement on *F*^2^	Primary atom site location: structure-invariant direct methods
Least-squares matrix: full	Secondary atom site location: difference Fourier map
*R*[*F*^2^ > 2σ(*F*^2^)] = 0.044	Hydrogen site location: inferred from neighbouring sites
*wR*(*F*^2^) = 0.112	H-atom parameters constrained
*S* = 1.05	*w* = 1/[σ^2^(*F*_o_^2^) + (0.0489*P*)^2^ + 0.2568*P*] where *P* = (*F*_o_^2^ + 2*F*_c_^2^)/3
4343 reflections	(Δ/σ)_max_ < 0.001
326 parameters	Δρ_max_ = 0.15 e Å^−^^3^
0 restraints	Δρ_min_ = −0.19 e Å^−^^3^

### Special details

**Table:**

Geometry. All esds (except the esd in the dihedral angle between two l.s. planes) are estimated using the full covariance matrix. The cell esds are taken into account individually in the estimation of esds in distances, angles and torsion angles; correlations between esds in cell parameters are only used when they are defined by crystal symmetry. An approximate (isotropic) treatment of cell esds is used for estimating esds involving l.s. planes.
Refinement. Refinement of F^2^ against ALL reflections. The weighted R-factor wR and goodness of fit S are based on F^2^, conventional R-factors R are based on F, with F set to zero for negative F^2^. The threshold expression of F^2^ > 2sigma(F^2^) is used only for calculating R-factors(gt) etc. and is not relevant to the choice of reflections for refinement. R-factors based on F^2^ are statistically about twice as large as those based on F, and R- factors based on ALL data will be even larger.

### Fractional atomic coordinates and isotropic or equivalent isotropic displacement parameters (Å^2^)

**Table:**

	*x*	*y*	*z*	*U*_iso_*/*U*_eq_	
C1	0.8725 (3)	0.2245 (2)	0.29320 (7)	0.0558 (5)	
H1A	1.0010	0.2303	0.2836	0.067*	
H1B	0.8028	0.2859	0.2828	0.067*	
C2	0.8736 (4)	0.2301 (2)	0.33899 (8)	0.0599 (6)	
H2A	0.9553	0.1741	0.3495	0.072*	
H2B	0.9236	0.2994	0.3474	0.072*	
C3	0.6797 (3)	0.21606 (19)	0.35581 (7)	0.0529 (5)	
H3	0.6016	0.2751	0.3455	0.063*	
C4	0.5845 (3)	0.10912 (18)	0.34382 (7)	0.0473 (5)	
C5	0.5903 (3)	0.10297 (16)	0.29712 (7)	0.0418 (4)	
H5	0.5147	0.1648	0.2883	0.050*	
C6	0.4890 (3)	0.00515 (18)	0.27942 (7)	0.0495 (5)	
H6A	0.5679	−0.0583	0.2822	0.059*	
H6B	0.3730	−0.0076	0.2941	0.059*	
C7	0.4445 (3)	0.02402 (17)	0.23522 (7)	0.0489 (5)	
H7A	0.3558	0.0833	0.2331	0.059*	
H7B	0.3836	−0.0399	0.2245	0.059*	
C8	0.6183 (3)	0.04992 (15)	0.20917 (7)	0.0427 (4)	
C9	0.7414 (3)	0.13761 (16)	0.23044 (6)	0.0423 (4)	
H9	0.6640	0.2031	0.2297	0.051*	
C10	0.7845 (3)	0.11993 (17)	0.27634 (7)	0.0438 (5)	
C11	0.9151 (3)	0.1655 (2)	0.20478 (7)	0.0563 (6)	
H11A	0.9845	0.2234	0.2177	0.068*	
H11B	0.9971	0.1030	0.2035	0.068*	
C12	0.8635 (3)	0.2003 (2)	0.16204 (7)	0.0543 (5)	
H12A	0.8006	0.2698	0.1630	0.065*	
H12B	0.9779	0.2089	0.1464	0.065*	
C13	0.7357 (3)	0.11911 (17)	0.14128 (7)	0.0458 (5)	
H13	0.8056	0.0509	0.1402	0.055*	
C14	0.5543 (3)	0.09684 (16)	0.16666 (7)	0.0424 (4)	
C15	0.4247 (3)	0.01587 (18)	0.14456 (7)	0.0519 (5)	
H15A	0.4808	−0.0554	0.1464	0.062*	
H15B	0.3048	0.0133	0.1585	0.062*	
C16	0.3869 (3)	0.0408 (2)	0.10007 (8)	0.0564 (5)	
H16A	0.3162	−0.0180	0.0881	0.068*	
H16B	0.3122	0.1061	0.0979	0.068*	
C17	0.5722 (3)	0.05540 (19)	0.07771 (7)	0.0523 (5)	
C18	0.6821 (3)	0.14719 (17)	0.09793 (7)	0.0479 (5)	
H18	0.5934	0.2076	0.0999	0.057*	
C19	0.8349 (3)	0.1842 (2)	0.06760 (7)	0.0553 (5)	
H19	0.9505	0.1429	0.0724	0.066*	
C20	0.8807 (4)	0.3028 (2)	0.06820 (8)	0.0686 (7)	
C21	0.7510 (4)	0.1508 (2)	0.02599 (8)	0.0705 (7)	
H21A	0.7358	0.2137	0.0090	0.085*	
H21B	0.8343	0.1002	0.0126	0.085*	
C22	0.5582 (4)	0.0977 (2)	0.03415 (8)	0.0651 (6)	
H22A	0.5354	0.0389	0.0155	0.078*	
H22B	0.4573	0.1501	0.0316	0.078*	
C23	0.3767 (3)	0.1168 (2)	0.35735 (8)	0.0648 (6)	
H23A	0.3143	0.1724	0.3423	0.097*	
H23B	0.3716	0.1338	0.3855	0.097*	
H23C	0.3152	0.0490	0.3527	0.097*	
C24	0.6753 (4)	0.0131 (2)	0.36544 (8)	0.0646 (6)	
H24A	0.6265	−0.0530	0.3546	0.097*	
H24B	0.6470	0.0168	0.3936	0.097*	
H24C	0.8094	0.0154	0.3617	0.097*	
C25	0.9274 (3)	0.0275 (2)	0.28368 (8)	0.0589 (6)	
H25A	0.9862	0.0372	0.3094	0.088*	
H25B	1.0217	0.0286	0.2630	0.088*	
H25C	0.8628	−0.0405	0.2832	0.088*	
C26	0.7285 (4)	−0.05744 (18)	0.20362 (8)	0.0577 (6)	
H26A	0.6720	−0.0987	0.1824	0.087*	
H26B	0.7244	−0.0982	0.2282	0.087*	
H26C	0.8571	−0.0418	0.1969	0.087*	
C27	0.4397 (3)	0.20241 (17)	0.17149 (7)	0.0496 (5)	
H27A	0.3419	0.1917	0.1910	0.074*	
H27B	0.3845	0.2217	0.1461	0.074*	
H27C	0.5217	0.2593	0.1803	0.074*	
C28	0.6745 (4)	−0.0533 (2)	0.07527 (7)	0.0577 (6)	
C29	1.0572 (6)	0.3352 (3)	0.07162 (10)	0.0984 (12)	
H29A	1.0859	0.4083	0.0703	0.118*	
H29B	1.1527	0.2848	0.0754	0.118*	
C30	0.7208 (7)	0.3804 (3)	0.06214 (16)	0.1213 (16)	
H30A	0.6549	0.3625	0.0378	0.182*	
H30B	0.7693	0.4526	0.0602	0.182*	
H30C	0.6358	0.3758	0.0845	0.182*	
C31	0.9582 (9)	−0.3072 (4)	0.0316 (2)	0.154 (2)	
H31A	0.8962	−0.2769	0.0086	0.232*	
H31B	0.8714	−0.3521	0.0461	0.232*	
H31C	1.0637	−0.3498	0.0230	0.232*	
O1	0.6889 (3)	0.22739 (17)	0.39892 (5)	0.0708 (5)	
H1	0.5933	0.2576	0.4070	0.106*	
O2	0.5943 (3)	−0.13820 (15)	0.07479 (8)	0.0919 (7)	
O3	0.8560 (3)	−0.04848 (17)	0.07166 (10)	0.0961 (8)	
H3A	0.8982	−0.1095	0.0683	0.144*	
O4	1.0176 (5)	−0.2288 (2)	0.05516 (11)	0.1310 (12)	
H4	1.1022	−0.2514	0.0697	0.197*	

### Atomic displacement parameters (Å^2^)

**Table:**

	*U*^11^	*U*^22^	*U*^33^	*U*^12^	*U*^13^	*U*^23^
C1	0.0482 (11)	0.0607 (13)	0.0586 (12)	−0.0167 (11)	0.0012 (11)	−0.0014 (11)
C2	0.0538 (12)	0.0635 (14)	0.0624 (13)	−0.0131 (11)	−0.0032 (12)	−0.0061 (12)
C3	0.0494 (11)	0.0542 (12)	0.0551 (12)	0.0044 (10)	−0.0024 (10)	−0.0023 (10)
C4	0.0382 (10)	0.0501 (11)	0.0536 (11)	0.0031 (9)	0.0006 (10)	0.0055 (10)
C5	0.0333 (9)	0.0362 (9)	0.0558 (11)	0.0030 (8)	−0.0002 (9)	0.0064 (9)
C6	0.0418 (10)	0.0434 (10)	0.0633 (13)	−0.0080 (9)	0.0052 (10)	0.0061 (10)
C7	0.0391 (9)	0.0451 (11)	0.0624 (12)	−0.0124 (9)	0.0012 (10)	0.0008 (10)
C8	0.0339 (9)	0.0364 (9)	0.0577 (12)	0.0011 (8)	0.0019 (9)	−0.0014 (9)
C9	0.0308 (9)	0.0417 (10)	0.0544 (11)	−0.0009 (8)	−0.0008 (9)	0.0001 (9)
C10	0.0313 (9)	0.0450 (10)	0.0552 (11)	0.0001 (9)	0.0003 (9)	0.0018 (9)
C11	0.0365 (10)	0.0771 (15)	0.0554 (12)	−0.0146 (11)	0.0012 (10)	0.0001 (12)
C12	0.0413 (10)	0.0671 (14)	0.0545 (12)	−0.0157 (10)	0.0043 (10)	0.0011 (11)
C13	0.0360 (9)	0.0471 (10)	0.0543 (11)	0.0006 (9)	0.0025 (9)	−0.0007 (10)
C14	0.0334 (8)	0.0382 (9)	0.0554 (11)	−0.0008 (8)	0.0023 (9)	0.0001 (9)
C15	0.0421 (10)	0.0515 (11)	0.0621 (13)	−0.0098 (9)	−0.0009 (10)	−0.0032 (11)
C16	0.0453 (11)	0.0591 (12)	0.0649 (13)	−0.0047 (10)	−0.0115 (11)	−0.0046 (11)
C17	0.0506 (11)	0.0522 (11)	0.0540 (12)	−0.0008 (10)	−0.0075 (10)	0.0000 (10)
C18	0.0441 (10)	0.0452 (11)	0.0543 (12)	−0.0007 (9)	0.0005 (10)	0.0002 (10)
C19	0.0563 (12)	0.0568 (12)	0.0528 (12)	−0.0060 (11)	0.0053 (11)	−0.0016 (11)
C20	0.0894 (19)	0.0612 (14)	0.0554 (13)	−0.0172 (15)	0.0160 (14)	−0.0033 (12)
C21	0.0819 (17)	0.0765 (16)	0.0532 (13)	−0.0124 (15)	0.0004 (14)	0.0000 (13)
C22	0.0743 (15)	0.0646 (14)	0.0564 (13)	−0.0050 (13)	−0.0122 (13)	0.0033 (12)
C23	0.0435 (11)	0.0870 (17)	0.0638 (14)	0.0008 (12)	0.0080 (11)	0.0005 (14)
C24	0.0694 (15)	0.0607 (14)	0.0638 (14)	0.0060 (12)	−0.0038 (13)	0.0145 (12)
C25	0.0409 (10)	0.0737 (15)	0.0621 (13)	0.0179 (11)	−0.0013 (11)	0.0006 (12)
C26	0.0615 (13)	0.0457 (11)	0.0659 (13)	0.0131 (11)	−0.0074 (12)	−0.0047 (11)
C27	0.0421 (10)	0.0464 (11)	0.0602 (12)	0.0072 (9)	0.0012 (10)	0.0027 (10)
C28	0.0654 (14)	0.0521 (13)	0.0555 (12)	−0.0026 (12)	0.0008 (11)	−0.0065 (11)
C29	0.127 (3)	0.100 (2)	0.0679 (17)	−0.058 (2)	−0.011 (2)	0.0139 (17)
C30	0.139 (3)	0.0624 (17)	0.162 (4)	0.013 (2)	0.058 (3)	0.019 (2)
C31	0.161 (5)	0.122 (4)	0.180 (5)	0.010 (4)	−0.045 (5)	−0.058 (4)
O1	0.0632 (10)	0.0922 (13)	0.0571 (9)	0.0064 (10)	0.0017 (8)	−0.0142 (9)
O2	0.1020 (15)	0.0548 (10)	0.1188 (17)	−0.0123 (11)	0.0369 (15)	−0.0120 (11)
O3	0.0664 (11)	0.0634 (10)	0.159 (2)	0.0099 (9)	−0.0085 (14)	−0.0236 (14)
O4	0.135 (2)	0.1133 (19)	0.144 (2)	0.0652 (18)	−0.068 (2)	−0.0608 (19)

### Geometric parameters (Å, º)

**Table:**

C1—C2	1.525 (3)	C16—H16B	0.9700
C1—C10	1.544 (3)	C17—C28	1.532 (3)
C1—H1A	0.9700	C17—C18	1.534 (3)
C1—H1B	0.9700	C17—C22	1.544 (4)
C2—C3	1.496 (3)	C18—C19	1.551 (3)
C2—H2A	0.9700	C18—H18	0.9800
C2—H2B	0.9700	C19—C20	1.505 (4)
C3—O1	1.443 (3)	C19—C21	1.563 (4)
C3—C4	1.540 (3)	C19—H19	0.9800
C3—H3	0.9800	C20—C29	1.320 (5)
C4—C24	1.532 (3)	C20—C30	1.501 (5)
C4—C23	1.545 (3)	C21—C22	1.542 (4)
C4—C5	1.556 (3)	C21—H21A	0.9700
C5—C6	1.527 (3)	C21—H21B	0.9700
C5—C10	1.557 (3)	C22—H22A	0.9700
C5—H5	0.9800	C22—H22B	0.9700
C6—C7	1.522 (3)	C23—H23A	0.9600
C6—H6A	0.9700	C23—H23B	0.9600
C6—H6B	0.9700	C23—H23C	0.9600
C7—C8	1.542 (3)	C24—H24A	0.9600
C7—H7A	0.9700	C24—H24B	0.9600
C7—H7B	0.9700	C24—H24C	0.9600
C8—C26	1.554 (3)	C25—H25A	0.9600
C8—C9	1.563 (3)	C25—H25B	0.9600
C8—C14	1.595 (3)	C25—H25C	0.9600
C9—C11	1.539 (3)	C26—H26A	0.9600
C9—C10	1.573 (3)	C26—H26B	0.9600
C9—H9	0.9800	C26—H26C	0.9600
C10—C25	1.549 (3)	C27—H27A	0.9600
C11—C12	1.531 (3)	C27—H27B	0.9600
C11—H11A	0.9700	C27—H27C	0.9600
C11—H11B	0.9700	C28—O2	1.196 (3)
C12—C13	1.521 (3)	C28—O3	1.295 (3)
C12—H12A	0.9700	C29—H29A	0.9300
C12—H12B	0.9700	C29—H29B	0.9300
C13—C18	1.532 (3)	C30—H30A	0.9600
C13—C14	1.564 (3)	C30—H30B	0.9600
C13—H13	0.9800	C30—H30C	0.9600
C14—C15	1.547 (3)	C31—O4	1.317 (6)
C14—C27	1.548 (3)	C31—H31A	0.9600
C15—C16	1.536 (4)	C31—H31B	0.9600
C15—H15A	0.9700	C31—H31C	0.9600
C15—H15B	0.9700	O1—H1	0.8200
C16—C17	1.522 (3)	O3—H3A	0.8200
C16—H16A	0.9700	O4—H4	0.8200
			
C2—C1—C10	113.8 (2)	C17—C16—C15	110.12 (18)
C2—C1—H1A	108.8	C17—C16—H16A	109.6
C10—C1—H1A	108.8	C15—C16—H16A	109.6
C2—C1—H1B	108.8	C17—C16—H16B	109.6
C10—C1—H1B	108.8	C15—C16—H16B	109.6
H1A—C1—H1B	107.7	H16A—C16—H16B	108.2
C3—C2—C1	111.3 (2)	C16—C17—C28	109.31 (19)
C3—C2—H2A	109.4	C16—C17—C18	108.26 (19)
C1—C2—H2A	109.4	C28—C17—C18	115.70 (19)
C3—C2—H2B	109.4	C16—C17—C22	116.3 (2)
C1—C2—H2B	109.4	C28—C17—C22	106.2 (2)
H2A—C2—H2B	108.0	C18—C17—C22	101.12 (19)
O1—C3—C2	108.62 (19)	C13—C18—C17	111.77 (18)
O1—C3—C4	111.2 (2)	C13—C18—C19	120.43 (18)
C2—C3—C4	114.0 (2)	C17—C18—C19	106.80 (18)
O1—C3—H3	107.6	C13—C18—H18	105.6
C2—C3—H3	107.6	C17—C18—H18	105.6
C4—C3—H3	107.6	C19—C18—H18	105.6
C24—C4—C3	111.19 (19)	C20—C19—C18	115.5 (2)
C24—C4—C23	108.2 (2)	C20—C19—C21	110.7 (2)
C3—C4—C23	106.88 (19)	C18—C19—C21	103.41 (19)
C24—C4—C5	114.82 (19)	C20—C19—H19	109.0
C3—C4—C5	106.82 (18)	C18—C19—H19	109.0
C23—C4—C5	108.60 (18)	C21—C19—H19	109.0
C6—C5—C4	114.31 (18)	C29—C20—C30	122.4 (3)
C6—C5—C10	110.66 (17)	C29—C20—C19	120.2 (3)
C4—C5—C10	117.39 (17)	C30—C20—C19	117.4 (3)
C6—C5—H5	104.3	C22—C21—C19	107.2 (2)
C4—C5—H5	104.3	C22—C21—H21A	110.3
C10—C5—H5	104.3	C19—C21—H21A	110.3
C7—C6—C5	110.40 (18)	C22—C21—H21B	110.3
C7—C6—H6A	109.6	C19—C21—H21B	110.3
C5—C6—H6A	109.6	H21A—C21—H21B	108.5
C7—C6—H6B	109.6	C21—C22—C17	104.6 (2)
C5—C6—H6B	109.6	C21—C22—H22A	110.8
H6A—C6—H6B	108.1	C17—C22—H22A	110.8
C6—C7—C8	114.14 (18)	C21—C22—H22B	110.8
C6—C7—H7A	108.7	C17—C22—H22B	110.8
C8—C7—H7A	108.7	H22A—C22—H22B	108.9
C6—C7—H7B	108.7	C4—C23—H23A	109.5
C8—C7—H7B	108.7	C4—C23—H23B	109.5
H7A—C7—H7B	107.6	H23A—C23—H23B	109.5
C7—C8—C26	106.96 (18)	C4—C23—H23C	109.5
C7—C8—C9	109.73 (17)	H23A—C23—H23C	109.5
C26—C8—C9	111.53 (16)	H23B—C23—H23C	109.5
C7—C8—C14	110.27 (16)	C4—C24—H24A	109.5
C26—C8—C14	110.46 (18)	C4—C24—H24B	109.5
C9—C8—C14	107.91 (15)	H24A—C24—H24B	109.5
C11—C9—C8	110.68 (18)	C4—C24—H24C	109.5
C11—C9—C10	114.46 (16)	H24A—C24—H24C	109.5
C8—C9—C10	116.83 (17)	H24B—C24—H24C	109.5
C11—C9—H9	104.4	C10—C25—H25A	109.5
C8—C9—H9	104.4	C10—C25—H25B	109.5
C10—C9—H9	104.4	H25A—C25—H25B	109.5
C1—C10—C25	107.34 (18)	C10—C25—H25C	109.5
C1—C10—C5	108.07 (18)	H25A—C25—H25C	109.5
C25—C10—C5	114.23 (18)	H25B—C25—H25C	109.5
C1—C10—C9	108.34 (17)	C8—C26—H26A	109.5
C25—C10—C9	112.54 (18)	C8—C26—H26B	109.5
C5—C10—C9	106.13 (16)	H26A—C26—H26B	109.5
C12—C11—C9	112.75 (18)	C8—C26—H26C	109.5
C12—C11—H11A	109.0	H26A—C26—H26C	109.5
C9—C11—H11A	109.0	H26B—C26—H26C	109.5
C12—C11—H11B	109.0	C14—C27—H27A	109.5
C9—C11—H11B	109.0	C14—C27—H27B	109.5
H11A—C11—H11B	107.8	H27A—C27—H27B	109.5
C13—C12—C11	112.25 (19)	C14—C27—H27C	109.5
C13—C12—H12A	109.2	H27A—C27—H27C	109.5
C11—C12—H12A	109.2	H27B—C27—H27C	109.5
C13—C12—H12B	109.2	O2—C28—O3	120.8 (3)
C11—C12—H12B	109.2	O2—C28—C17	123.2 (2)
H12A—C12—H12B	107.9	O3—C28—C17	115.8 (2)
C12—C13—C18	115.19 (18)	C20—C29—H29A	120.0
C12—C13—C14	111.25 (18)	C20—C29—H29B	120.0
C18—C13—C14	110.13 (17)	H29A—C29—H29B	120.0
C12—C13—H13	106.6	C20—C30—H30A	109.5
C18—C13—H13	106.6	C20—C30—H30B	109.5
C14—C13—H13	106.6	H30A—C30—H30B	109.5
C15—C14—C27	106.52 (17)	C20—C30—H30C	109.5
C15—C14—C13	110.32 (17)	H30A—C30—H30C	109.5
C27—C14—C13	109.85 (16)	H30B—C30—H30C	109.5
C15—C14—C8	110.77 (16)	O4—C31—H31A	109.5
C27—C14—C8	111.42 (17)	O4—C31—H31B	109.5
C13—C14—C8	107.97 (15)	H31A—C31—H31B	109.5
C16—C15—C14	115.59 (19)	O4—C31—H31C	109.5
C16—C15—H15A	108.4	H31A—C31—H31C	109.5
C14—C15—H15A	108.4	H31B—C31—H31C	109.5
C16—C15—H15B	108.4	C3—O1—H1	109.5
C14—C15—H15B	108.4	C28—O3—H3A	109.5
H15A—C15—H15B	107.4	C31—O4—H4	109.5
			
C10—C1—C2—C3	−55.7 (3)	C18—C13—C14—C27	−67.3 (2)
C1—C2—C3—O1	−177.2 (2)	C12—C13—C14—C8	−60.0 (2)
C1—C2—C3—C4	58.2 (3)	C18—C13—C14—C8	171.05 (17)
O1—C3—C4—C24	−51.5 (2)	C7—C8—C14—C15	−57.9 (2)
C2—C3—C4—C24	71.7 (3)	C26—C8—C14—C15	60.2 (2)
O1—C3—C4—C23	66.4 (2)	C9—C8—C14—C15	−177.70 (16)
C2—C3—C4—C23	−170.4 (2)	C7—C8—C14—C27	60.5 (2)
O1—C3—C4—C5	−177.52 (17)	C26—C8—C14—C27	178.54 (17)
C2—C3—C4—C5	−54.3 (2)	C9—C8—C14—C27	−59.3 (2)
C24—C4—C5—C6	60.3 (2)	C7—C8—C14—C13	−178.75 (16)
C3—C4—C5—C6	−175.89 (17)	C26—C8—C14—C13	−60.7 (2)
C23—C4—C5—C6	−60.9 (2)	C9—C8—C14—C13	61.41 (19)
C24—C4—C5—C10	−71.7 (2)	C27—C14—C15—C16	70.8 (2)
C3—C4—C5—C10	52.0 (2)	C13—C14—C15—C16	−48.4 (2)
C23—C4—C5—C10	166.98 (19)	C8—C14—C15—C16	−167.85 (18)
C4—C5—C6—C7	161.11 (17)	C14—C15—C16—C17	53.2 (3)
C10—C5—C6—C7	−63.7 (2)	C15—C16—C17—C28	68.7 (2)
C5—C6—C7—C8	56.8 (2)	C15—C16—C17—C18	−58.1 (2)
C6—C7—C8—C26	74.0 (2)	C15—C16—C17—C22	−171.1 (2)
C6—C7—C8—C9	−47.1 (2)	C12—C13—C18—C17	173.50 (19)
C6—C7—C8—C14	−165.83 (17)	C14—C13—C18—C17	−59.7 (2)
C7—C8—C9—C11	−179.51 (18)	C12—C13—C18—C19	47.0 (3)
C26—C8—C9—C11	62.2 (2)	C14—C13—C18—C19	173.75 (18)
C14—C8—C9—C11	−59.3 (2)	C16—C17—C18—C13	63.8 (2)
C7—C8—C9—C10	47.1 (2)	C28—C17—C18—C13	−59.2 (3)
C26—C8—C9—C10	−71.2 (2)	C22—C17—C18—C13	−173.45 (19)
C14—C8—C9—C10	167.28 (16)	C16—C17—C18—C19	−162.52 (18)
C2—C1—C10—C25	−73.5 (3)	C28—C17—C18—C19	74.4 (2)
C2—C1—C10—C5	50.1 (3)	C22—C17—C18—C19	−39.8 (2)
C2—C1—C10—C9	164.69 (19)	C13—C18—C19—C20	−85.4 (3)
C6—C5—C10—C1	175.72 (18)	C17—C18—C19—C20	145.8 (2)
C4—C5—C10—C1	−50.6 (2)	C13—C18—C19—C21	153.6 (2)
C6—C5—C10—C25	−64.9 (2)	C17—C18—C19—C21	24.8 (2)
C4—C5—C10—C25	68.8 (2)	C18—C19—C20—C29	128.0 (3)
C6—C5—C10—C9	59.7 (2)	C21—C19—C20—C29	−114.9 (3)
C4—C5—C10—C9	−166.61 (17)	C18—C19—C20—C30	−55.8 (4)
C11—C9—C10—C1	59.3 (2)	C21—C19—C20—C30	61.3 (4)
C8—C9—C10—C1	−169.06 (17)	C20—C19—C21—C22	−124.1 (2)
C11—C9—C10—C25	−59.3 (2)	C18—C19—C21—C22	0.2 (3)
C8—C9—C10—C25	72.4 (2)	C19—C21—C22—C17	−24.6 (3)
C11—C9—C10—C5	175.12 (18)	C16—C17—C22—C21	155.9 (2)
C8—C9—C10—C5	−53.2 (2)	C28—C17—C22—C21	−82.2 (2)
C8—C9—C11—C12	55.0 (3)	C18—C17—C22—C21	38.9 (3)
C10—C9—C11—C12	−170.47 (19)	C16—C17—C28—O2	28.9 (3)
C9—C11—C12—C13	−52.4 (3)	C18—C17—C28—O2	151.4 (3)
C11—C12—C13—C18	−178.50 (19)	C22—C17—C28—O2	−97.3 (3)
C11—C12—C13—C14	55.3 (3)	C16—C17—C28—O3	−154.5 (3)
C12—C13—C14—C15	178.84 (18)	C18—C17—C28—O3	−32.0 (4)
C18—C13—C14—C15	49.9 (2)	C22—C17—C28—O3	79.3 (3)
C12—C13—C14—C27	61.7 (2)		

### Hydrogen-bond geometry (Å, º)

**Table:**

*D*—H···*A*	*D*—H	H···*A*	*D*···*A*	*D*—H···*A*
O3—H3*A*···O4	0.82	1.76	2.571 (3)	170
O1—H1···O2^i^	0.82	1.95	2.753 (3)	165
O4—H4···O1^ii^	0.82	1.83	2.640 (3)	168

Symmetry codes: (i) −*x*+1, *y*+1/2, −*z*+1/2; (ii) −*x*+2, *y*−1/2, −*z*+1/2.
