# Supplementary figures and images for: Crystal structure of betulinic acid methanol monosolvate
Source: Acta Crystallogr Sect E Struct Rep Online. 2014 Nov 8;70(Pt 12):o1242–3. doi: 10.1107/S1600536814023848 (PMC4257419; doi:10.1107/S1600536814023848)

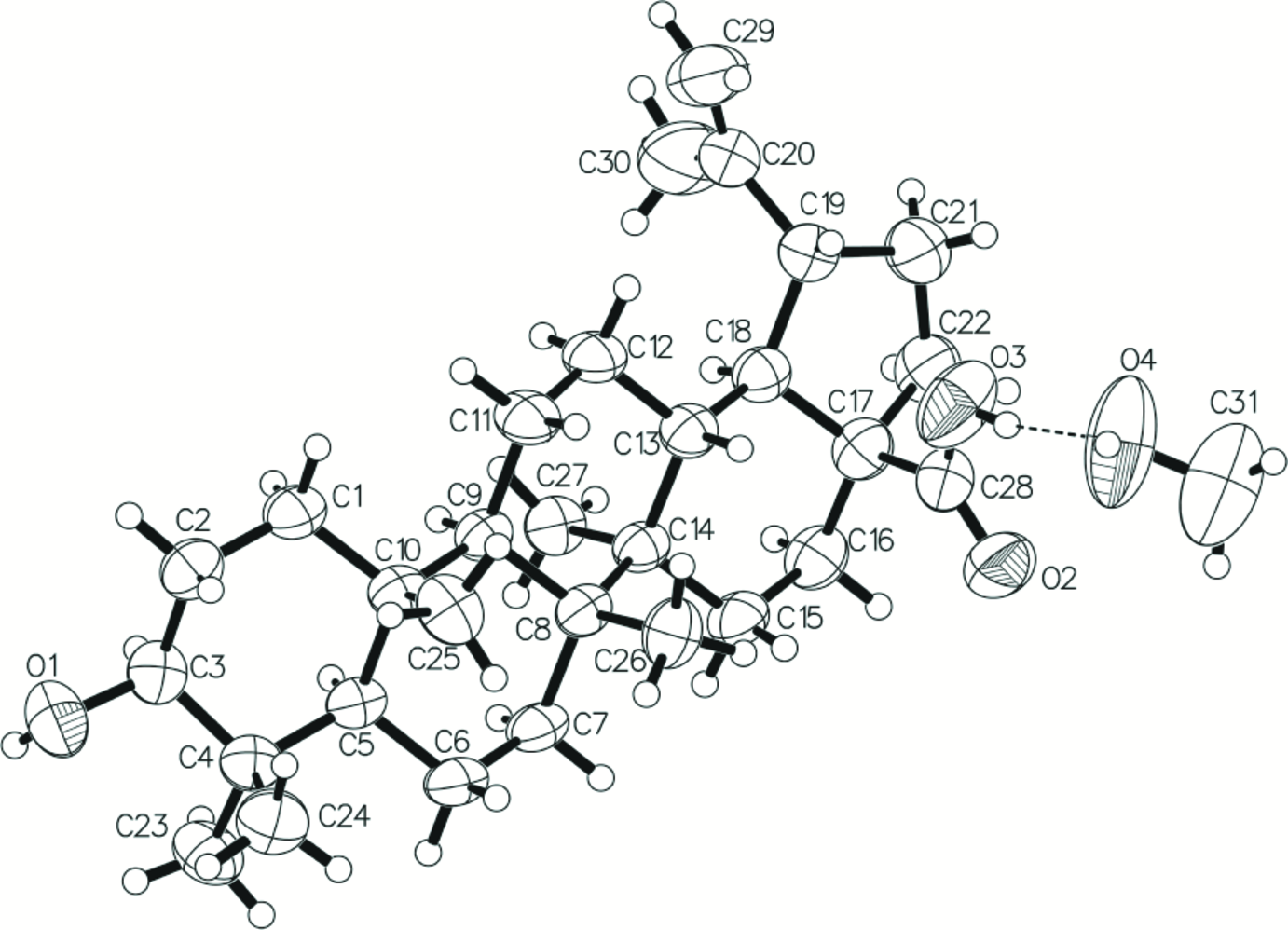

Supplement: Supplementary file 3 [file e-70-o1242-fig1.tif]
